# Trigger Finger: An Unusual Clue to Hashimoto’s Thyroiditis

**DOI:** 10.7759/cureus.71143

**Published:** 2024-10-09

**Authors:** Noah Llaneras, Valeria Ross, Roberto Miki

**Affiliations:** 1 Orthopedic Surgery/Plastic and Reconstructive Surgery, Herbert Wertheim College of Medicine, Florida International University, Miami, USA; 2 Orthopedic Surgery, Herbert Wertheim College of Medicine, Florida International University, Miami, USA

**Keywords:** autoimmune disease, orthopaedic hand surgery, patient safety based medical education, trigger finger disorder, hashimoto’s thyroiditis

## Abstract

Stenosing flexor tenosynovitis, commonly known as trigger finger (TF), is characterized by thickening and inflammation of the flexor tendon sheath (A1 pulley), leading to painful catching or locking of the finger in a flexed position. While often associated with conditions like diabetes and hypothyroidism, this case report presents a unique instance where surgical intervention for pharmacologically resistant TF ultimately led to the diagnosis of Hashimoto's thyroiditis. This case highlights the potential for underlying systemic conditions to manifest as TF and emphasizes the importance of a comprehensive diagnostic approach in patients with persistent or atypical presentations.

## Introduction

Trigger finger (TF) is a prevalent hand tendinopathy affecting an estimated 2% of the population [1]. Characterized by discomfort, swelling, and impaired hand function, tendinopathy encompasses a range of clinical presentations [2]. TF typically affects adults in a bimodal distribution, with peak incidences in the fifth and sixth decades of life [3]. Treatment options vary in invasiveness, ranging from conservative measures like splinting, therapy, and anti-inflammatory medications to corticosteroid injections and surgical release (both open and percutaneous). Notably, TF is often associated with systemic diseases, including type 1 diabetes, hypothyroidism, and rheumatological disorders.

Emerging evidence suggests a link between musculoskeletal symptoms, including TF, and underlying thyroid disease [4]. Thyroid hormone receptors, present in healthy hand tendons, are thought to play a role in extracellular matrix regulation, potentially influencing collagen production and tendon health. Fluctuations in triiodothyronine (T3) and thyroxine (T4) levels, characteristic of thyroid dysfunction, can disrupt the delicate balance of collagen matrix and tenocyte activity, contributing to the development of tendinopathy. However, the precise pathophysiological mechanisms underlying the association between thyroid dysfunction and TF remain to be fully elucidated [2,5].

We present a unique case of a patient who initially presented with TF symptoms and was ultimately diagnosed with Hashimoto's thyroiditis.

## Case presentation

**Figure 1:**
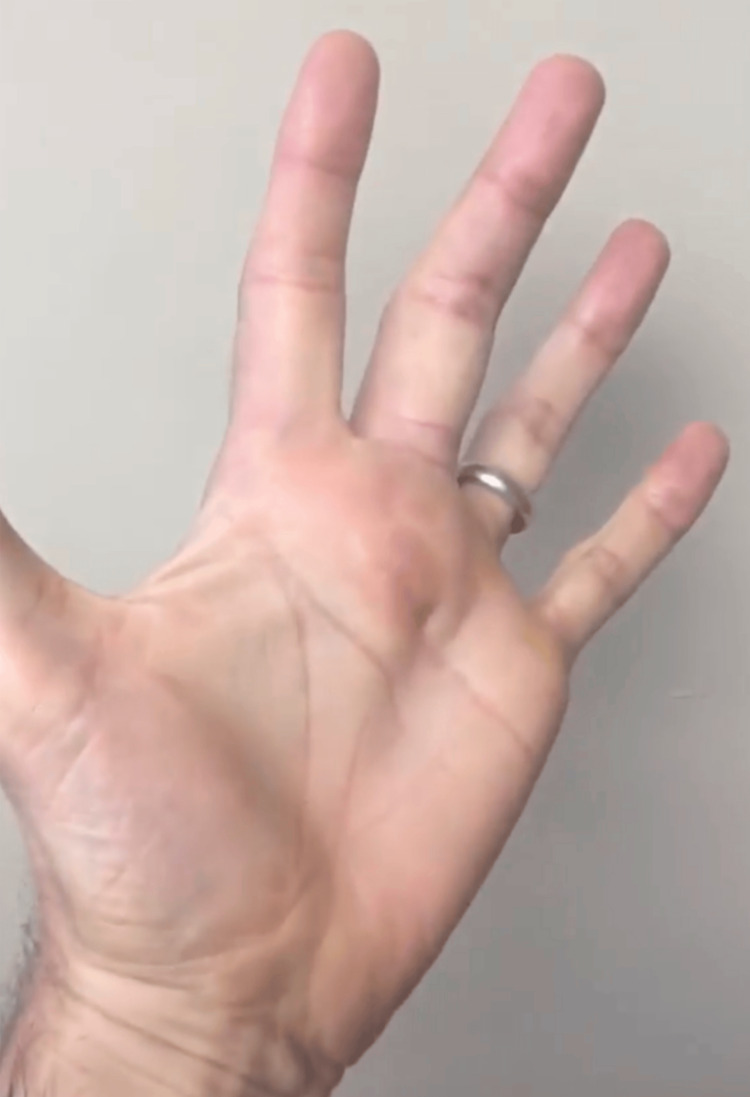
Anterior view of the left hand Preoperative clinical photo of the patient’s left hand showing swelling localized over the A1 pulley of the left long finger.

**Figure 2:**
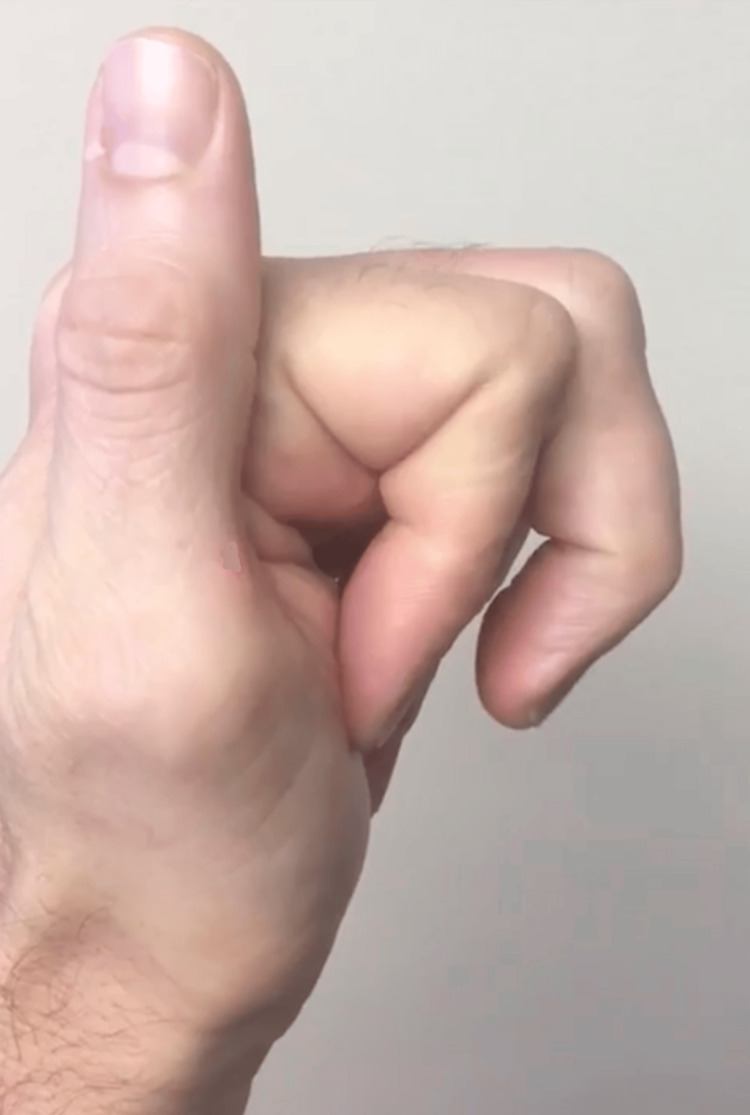
Sagittal view of the left hand Preoperative clinical photo of the patient’s left hand. In this sagittal view, the patient is unable to fully flex the long finger.

A 39-year-old right-hand dominant male orthopedic surgeon presented with a nine-month history of pain and swelling at the left long finger A1 pulley (Figures 1, 2). The catching and locking in his left hand progressively worsened, making it increasingly difficult to perform daily activities. He denied any puncture wounds to the area and had no significant past medical history. However, he did report injuring his finger while positioning a patient on the operating room table just before the onset of his symptoms.

**Figure 3:**
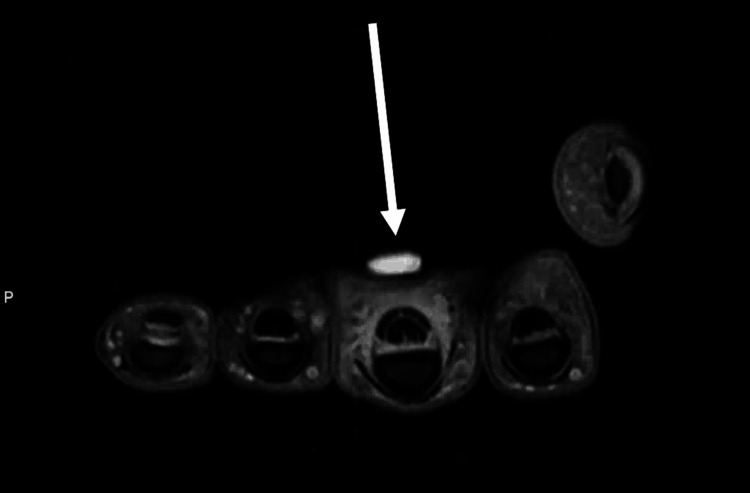
Preoperative MRI axial image of the left hand There was noticeable swelling in the left long finger flexor tendon sheath.

Before presenting to our clinic, the patient received three corticosteroid injections to alleviate pain and address symptoms. While these injections provided temporary relief, his symptoms consistently returned after three months. MRI of the left hand revealed fluid in the flexor tendon sheath and possible A2 pulley attenuation (Figure 3). The patient's presentation, particularly the prolonged duration of symptoms and functional impairment, raised concerns for potential rheumatological conditions such as rheumatoid arthritis or connective tissue disorders. To investigate this possibility, laboratory testing was conducted, including a complete blood count, basic metabolic panel, cyclic citrullinated peptide antibodies, rheumatoid factor, antinuclear antibodies, erythrocyte sedimentation rate, Lyme titer, and C-reactive protein. All results were within normal limits, suggesting a lower likelihood of a primary rheumatological disorder.

Given the persistent symptoms despite non-operative management, surgical intervention was recommended. The patient's prolonged history of symptoms and lack of response to conservative treatment, in the absence of clear traumatic etiology, raised suspicion for an atypical infectious process. To ensure uncontaminated cultures, surgery was performed without preoperative antibiotics, allowing for optimal microbial growth detection. Under local anesthesia and sedation, a left long finger flexor tenosynovectomy and A1 pulley release were performed after exsanguination of the left upper extremity from the wrist to the elbow. Intraoperatively, extensive synovitis was noted proximal to the A1 pulley, restricting full finger extension. The flexor digitorum superficialis and profundus tendons were also encased in tenosynovitis. All synovial tissue and fluid were sent for cultures (aerobic, anaerobic, acid-fast bacillus, and fungal) and pathological analysis. The patient was prescribed a short course of doxycycline postoperatively. All cultures remained negative, and pathology revealed synovium, dense regular connective tissue, and soft tissue with chronic inflammation, focal fibrin deposition, and histiocyte response.

Six months postoperatively, the patient experienced recurrent palmar swelling in his left hand, accompanied by clicking of the left long finger. He could not make a full fist or fully extend the proximal interphalangeal joint. Given the recurrence of symptoms, a broad diagnostic approach was taken to explore potential systemic contributors. This included ordering a Lyme titer to rule out Lyme disease, a condition known to cause joint inflammation, and thyroid-stimulating hormone levels to assess for hypothyroidism, which can manifest with joint symptoms and soft tissue swelling [6]. The patient's TSH level was elevated at 6.2 uIU/mL (normal range 0.4-4.0 uIU/mL). Due to the persistent and recurrent nature of his symptoms, a repeat exploration, culture, and biopsy were performed.

The patient underwent a second surgery under regional anesthesia of the median and radial nerves at the wrist. Upon reopening the previous incision, a significant amount of tenosynovitis was observed. A tenosynovectomy of the flexor tendons was performed, and synovial fluid was sent for cultures and pathology, including acid-fast and fungal stains. Intraoperatively, the finger demonstrated full flexion and extension without clicking.

Pathology again revealed chronic fibrinous synovitis with no granulomas. Acid-fast bacillus and GMS (fungal) stains were negative. Given the patient's previously elevated TSH, further investigation was warranted. Elevated thyroid peroxidase antibody levels (157 IU/mL and 1532 IU/mL) were found, contributing to a diagnosis of Hashimoto's thyroiditis. The diagnosis is typically made based on the presence of elevated TSH levels, low levels of free T4, and positive thyroid peroxidase antibodies [7]. This patient exhibited all three criteria, confirming the diagnosis.

Following his diagnosis, the patient was treated with synthetic T4 and levothyroxine for hypothyroidism. Five years after surgical intervention, Hashimoto's thyroiditis remains well-controlled, and he has not experienced any recurrence of TF symptoms.

## Discussion

This case highlights an atypical presentation of TF, a hand disorder affecting 28 out of every 100000 individuals [8]. The patient's unusual clinical course ultimately led to a diagnosis of Hashimoto's thyroiditis, emphasizing the importance of a broad differential diagnosis in persistent or recurrent cases. Physicians should maintain a high index of suspicion for such conditions, even when presenting symptoms are primarily musculoskeletal.

This case presents a rare instance of chronic stenosing flexor tenosynovitis as the presenting symptom of thyroid disease. The atypical nature of this presentation, specifically the recurrence of TF in the same digit following surgical release, warranted a more extensive evaluation. In such cases, a broad differential diagnosis must be considered, encompassing possibilities such as infection, atypical infection (mycobacterial and fungal), thyroid disease, diabetes, and rheumatological/inflammatory disorders. A rheumatoid panel, including a thyroid-stimulating hormone level, was obtained in this case and should be considered in all atypical presentations of TF to uncover potentially treatable underlying conditions [9].

Atypical infections, such as fungal and mycobacterial infections of the flexor tendon sheath, are uncommon but should remain on the differential, particularly in patients with relevant occupational or recreational exposures [10]. While our patient had no history of marine or bird exposure, the possibility of contamination from previous steroid injections was considered. However, cultures and staining from both surgical procedures were negative for mycobacterial and fungal infections.

The elevated TSH level revealed by the rheumatoid panel aided in the diagnosis of Hashimoto's thyroiditis, an autoimmune disease characterized by the gradual destruction of the thyroid gland [7]. While genetic predisposition plays a role, environmental factors like amiodarone, lithium, and iodine exposure can trigger the disease [11]. This patient did not present with any of these predisposing risk factors. Hashimoto's thyroiditis typically presents with thyroid gland enlargement (goiter) in its early stages. As the disease progresses, the thyroid gland shrinks, and thickening and scarring of the connective tissues occur due to the destruction of thyroid follicles [7]. This process often leads to hypothyroidism, a condition in which the thyroid gland doesn't produce enough hormones [12]. Approximately one in 10 women will develop Hashimoto's thyroiditis [13]. Management typically involves monitoring for signs of hypothyroidism and initiating T4 replacement therapy when necessary to restore hormone balance and alleviate symptoms [14].

In this case, the patient's recurrent TF proved resistant to both non-operative and operative treatments. Extensive workup, including blood tests, intraoperative cultures, and pathological analysis, ruled out other potential diagnoses, such as infection, atypical infection, type 1 diabetes, and rheumatological/inflammatory disorders. The diagnosis of Hashimoto's hypothyroidism and subsequent treatment with levothyroxine successfully resolved the patient's recurrent TF.

## Conclusions

This case underscores the importance of considering underlying systemic conditions in patients with TF, particularly when presentations are atypical or refractory to standard treatments (including surgery). While stenosing tenosynovitis, characterized by fibrocartilaginous metaplasia of the tendon sheath at the A1 pulley, often presents idiopathically, this case highlights the potential role of hypothyroidism in its development. Our patient's recurrent TF, resistant to both conservative and surgical management, ultimately led to the diagnosis of Hashimoto's thyroiditis. This case emphasizes the need for a comprehensive evaluation, including assessment for underlying metabolic and endocrine disorders, in patients with TF, especially when clinical suspicion dictates further investigation.
